# Compositional mantle layering revealed by slab stagnation at ~1000-km depth

**DOI:** 10.1126/sciadv.1500815

**Published:** 2015-12-10

**Authors:** Maxim D. Ballmer, Nicholas C. Schmerr, Takashi Nakagawa, Jeroen Ritsema

**Affiliations:** 1 Earth-Life Science Institute, Tokyo Institute of Technology, Tokyo 152-8551, Japan.; 2 School of Ocean and Earth Sciences and Technology, University of Hawaii at Manoa, Honolulu, HI 96822, USA.; 3 Department of Geology, University of Maryland, College Park, MD 20742, USA.; 4 Department of Mathematical Science and Advanced Technology, Japan Agency for Marine-Earth Science and Technology, Yokohama 236-0001, Japan.; 5 Department of Earth and Environmental Sciences, University of Michigan, Ann Arbor, MI 48109, USA.

**Keywords:** mantle convection, mantle composition, subducted slab, stagnant slab, bulk silicate Earth

## Abstract

Improved constraints on lower-mantle composition are fundamental to understand the accretion, differentiation, and thermochemical evolution of our planet. Cosmochemical arguments indicate that lower-mantle rocks may be enriched in Si relative to upper-mantle pyrolite, whereas seismic tomography images suggest whole-mantle convection and hence appear to imply efficient mantle mixing. This study reconciles cosmochemical and geophysical constraints using the stagnation of some slab segments at ~1000-km depth as the key observation. Through numerical modeling of subduction, we show that lower-mantle enrichment in intrinsically dense basaltic lithologies can render slabs neutrally buoyant in the uppermost lower mantle. Slab stagnation (at depths of ~660 and ~1000 km) and unimpeded slab sinking to great depths can coexist if the basalt fraction is ~8% higher in the lower mantle than in the upper mantle, equivalent to a lower-mantle Mg/Si of ~1.18. Global-scale geodynamic models demonstrate that such a moderate compositional gradient across the mantle can persist can in the presence of whole-mantle convection.

## Teaser

The stagnation of ~1000-km deep slabs indicates that dense basalt may be more abundant in the lower mantle than in the upper mantle.

## INTRODUCTION

Earth’s upper-mantle composition can be estimated from mid–ocean ridge basalt (MORB) geochemistry (*1*, *2*), whereas the lower mantle remains poorly understood. Geophysical observations have been directly compared to laboratory measurements of rock properties at lower-mantle pressures and temperatures, but results drawn from such efforts remain not unique (*3*–*9*). Cosmochemical arguments for chondritic bulk Earth compositions imply that lower-mantle rocks are enriched in Si relative to upper-mantle pyrolite (*10*, *11*), unless the core contains ≥7% Si (*12*). Instead, seismic observations of the subducted Tethys and Farallon lithosphere in the lowermost mantle attest to whole-mantle convection and stirring (*13*–*17*) and may thus point toward a pyrolitic whole mantle (*1*, *2*). Mantle mixing is predicted to be efficient despite the impedance of mantle flow across the endothermic wadsleyite-to-bridgmanite phase transition at 660-km depth (hereinafter, the 660) (*18*).

**Fig. 1:**
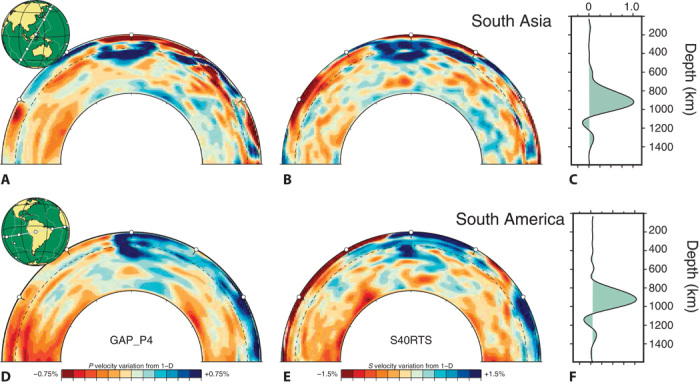
Seismic evidence for slab stagnation in the uppermost lower mantle. Cross sections through the *P*-wave model GAP-P4 (*16*) (**A** and **D**) and the *S*-wave model S40RTS (*15*) (**B** and **E**) centered on South Asia (top row) and South America (bottom row). (**C** and **F**) Resolution kernels quantify the depth range, over which the S40RTS *S*-wave velocity structure is mapped for a hypothetical point anomaly at 900 km. These kernels demonstrate that flat slabs imaged in the uppermost lower mantle beneath Peru and Indonesia are well resolved (that is, independently resolved from MTZ heterogeneity).

However, seismic tomography reveals that only a subset of slabs readily sinks into the deep lower mantle (*13*–*17*). Some slabs stagnate in the mantle transition zone (MTZ) for tens of millions of years, such as beneath Europe, East Asia, and North America. Slab stagnation above the 660 is explained by the combined effects of the endothermic phase transition and associated viscosity jump, particularly if the trench retreats and slabs roll back through the mantle, a configuration that reduces slab dip and maximizes the area of slab-660 interaction (*19*–*21*). Other slab segments that are able to cross the “barrier” at the 660, however, flatten at slightly greater depths (for example, Peru, Sunda, Mexico, Kuriles, and Kermadec) (*16*). These observations from recent *P*-wave tomography (*16*) are confirmed by independent *S*-wave tomography (*15*) with a good resolution in the uppermost lower mantle (Fig. 1). In contrast to slab stagnation above the 660, slab flattening in the uppermost lower mantle, where no major phase transitions are thought to occur, remains poorly understood.

Here, we test the hypothesis that subducted slabs become neutrally buoyant and stagnate in equilibrium at ~1000-km depth if the lower mantle is enriched in some intrinsically dense lithology; we postulate that subducted MORB (and high-pressure polymorphs) is the most straightforward candidate. We investigate the effects of lower-mantle basalt enrichment on slab descent by running a series of regional high-resolution numerical experiments. Finally, we demonstrate that a gradual increase of basalt concentration across the MTZ can be sustained over billions of years of whole-mantle convection using global-scale geodynamic models.

## RESULTS

**Fig. 2:**
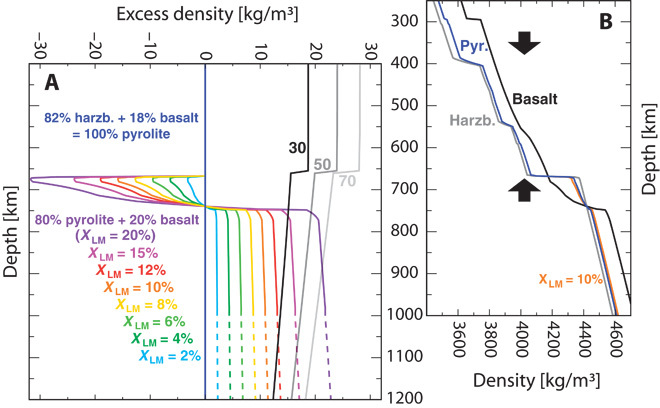
Density of subducted slabs and ambient mantle as a function of *X*_LM_. (**A**) Semianalytical solution for the density of subducted slabs relative to the ambient mantle. The black-to-gray curves show the thermal density anomaly of subducted slabs of ages at the trench as annotated (in millions of years) relative to pyrolitic mantle (blue reference curve). Thermal density anomalies are averages over the cool slab core of thickness 250 km, calculated using a parameterization for depth-dependent thermal expansivity and accounting for thermal diffusion during slab sinking (see “Methods for slab-sinking models”). Slab-sinking speeds in the upper and lower mantle are assumed to be 60 and 6 km/My, respectively (*52*). Any compositional density difference between the slab and pyrolite is ignored because of a density trade-off between the slab’s basaltic and harzburgitic domains. For comparison, colored curves show densities (relative to pyrolite) of lower mantle compositions enriched in basalt (see table S3). Dashed lines are extrapolations. (**B**) Colored curves are calculated from the absolute density profiles of mantle materials [taken from Xu *et al.* (*22*)]. Black arrows mark depth ranges, in which basalt is strongly negatively or positively buoyant.

Density profiles derived from mineral-physics databases (*22*) robustly demonstrate that the density of sinking slabs can be similar to that of a lower mantle enriched in basalt (Fig. 2). Slab density is a function of its age τ during subduction, and lower-mantle density is a function of its average basalt fraction *X*_LM_. In the upper mantle, which should have a similar bulk composition to the slab, and above the post–garnet phase transition at ~750-km depth (*23*), the cool slabs are negatively buoyant and sink. In the lower mantle, they may become near–neutrally buoyant and stagnate for 8% ≤ *X*_LM_ ≤ 20% (black-to-gray versus yellow-to-purple curves in Fig. 2A).

**Fig. 3:**
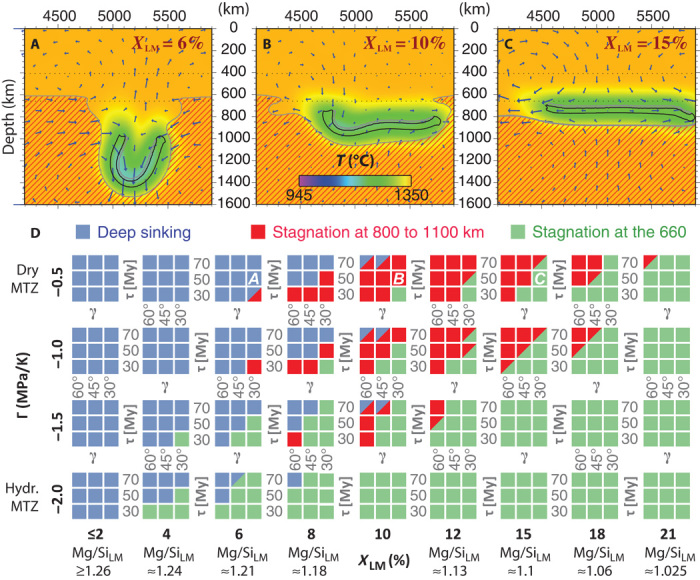
Numerical-model predictions for three regimes of slab-sinking behavior. (**A** to **C**) Arrows denote velocity vectors and colors indicate potential temperature in snapshots at 150 My (for time series, see fig. S3). The 5% iso-contours of harzburgite (black) and basalt fraction (gray) are labeled. Domains with *X*_LM_ > 0 are shown with dark red hatching. Slab-sinking behavior varies as a function of *X*_LM_ (as annotated) between the cases shown. (**D**) Map of slab-sinking regimes as a function of all parameters. Mantle parameters Γ and *X*_LM_ are varied between big squares; slab parameters τ and β are varied between small squares (that is, within each big square). Cases (A to C) are labeled. For Γ = −3 MPa/K, slabs are always predicted to stagnate at the 660 (not shown). Bottom scale: Mg/Si of the lower mantle, calculated from *X*_LM_ [according to Workman and Hart (*2*)].

To quantify the effects of *X*_LM_ on slab dynamics, we explore two-dimensional numerical models of slab sinking through the mantle (Fig. 3) (see “Methods for slab-sinking models ”). We simulate the compositional difference between the upper and lower mantle by imposing that basaltic heterogeneity is initially more abundant (by *X*_LM_) below than above the 660 (*X*_LM_ = 0% for pyrolite; see table S3). The sharp transition in composition across the 660 in our model is a simplification; is a simplification; basalt distribution in the Earth’s mantle reflects the history of basalt extraction and subduction (see below). In addition to parameters *X*_LM_ and τ, we vary the Clapeyron slope Γ of the 660 and the initial slab angle β.

Three different regimes of slab behavior are manifested (Fig. 3; see “Sinking behavior of slab segments”). In regime I, the slab decelerates and deforms as it penetrates the 660 but ultimately sinks into the deep mantle. In regime II, the slab segment also penetrates the 660 but stagnates at depths of about 800 to 1050 km. Here, the slab has become near–neutrally buoyant and spreads horizontally or subhorizontally for ~500 million years (My) or longer. In regime III, the slab fails to penetrate the 660 and flattens above it. Regimes I and III have been described previously by Christensen and Yuen (*19*); regime II is described here for the first time.

Figure 3D shows that slab behavior depends on “slab parameters” (τ and β) and “mantle parameters” (Γ and *X*_LM_). Penetration of the 660 is enhanced by decreasing absolute Γ and/or increasing β. Steeper slabs tend to penetrate because they exert a higher stress on the 660 (that is, at any given area of 660 depression). Instead, higher absolute Γ values make the 660 less permeable by enhancing 660 topography (*19*). Moreover, the density contrast between the slab and the lower mantle as a function of *X*_LM_ and τ (Fig. 2A) determines whether slabs sink deep into the mantle. Slab descent is facilitated for relatively low values of *X*_LM_ and high values of τ.

In addition to variations of slab age and angle, mantle compositional heterogeneity suitably accounts for the coexistence of the three regimes of slab behavior in the modern Earth. For example, slabs may not readily penetrate the 660 in regions with a high transition–zone water content [on which Γ is dependent (*24*)] or stagnate at ~1000-km depth if locally high *X*_LM_ promotes buoyancy (for example, Fig. 3, A versus B). Sensitivity of slab behavior to mantle heterogeneity may explain why some, but not all, young slab segments (for example, Nicaragua) sink whereas some, but not all, old slab segments (for example, Japan) stagnate at the 660 [cf. King *et al.* (*21*) and Karato *et al.* (*25*) for alternative explanations]. Mantle heterogeneity may further explain why part of the Farallon slab stagnates beneath North America, whereas most of it sinks deep into the lower mantle beneath the Atlantic.

The sensitivity of slab-sinking behavior on mantle composition can provide constraints for Earth’s average *X*_LM_ and Γ. Figure 3D shows that all three regimes of slab behavior can only coexist simultaneously for *X*_LM_ ≈ 8% and Γ = −1.0 ± 0.5 MPa/K. These values correspond to an average lower-mantle perovskite content of ~87%, or Mg/Si ≈ 1.18, about halfway between pyrolitic and perovskitic compositions (table S3), and a dry to moderately hydrated MTZ (*24*). We note, however, that the challenge to quantify *X*_LM_ on the basis of the behavior of slabs that sink through a convecting heterogeneous mantle implies significant uncertainty for these estimates (of at least a couple of percentages). Nevertheless, on the basis of seismic observations, we can reject pyrolitic (*X*_LM_ = 0%) and perovskitic (*X*_LM_ ≈ 15 to 20%, that is, corresponding to chondritic Mg/Si) end-member lower-mantle compositions because they rule out slab stagnation in the uppermost lower mantle and deep sinking, respectively. The coexistence of three distinct slab-sinking regimes (*13*–*16*) indeed points to a moderate compositional difference between the upper and lower mantle, possibly originating from core-mantle differentiation or fractional crystallization of the magma ocean (*26*, *27*).

Independent of the origin, however, mechanisms to maintain, or even create, mantle compositional layering over >4 billion years (Gy) of whole-mantle convective stirring are needed. Basalt tends to concentrate in the MTZ, because its density anomaly relative to pyrolite peaks at 300 to 410 km and is reversed at depths of 660 to 740 km (*28*) (arrows in Fig. 2B). However, because the depth range of the density reversal is narrow (*23*), basalt may ultimately enter the lower mantle to be dispersed and to accumulate there.

**Fig. 4:**
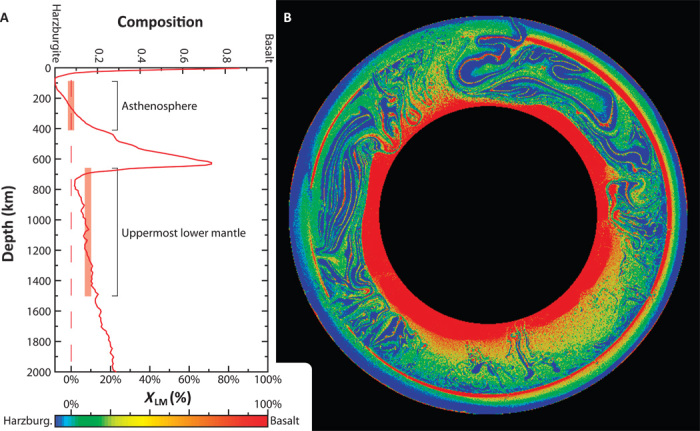
Compositional mantle layering predicted by a global-scale thermochemical mantle-convection model (case A1) after ~4.57 Gy model time. (**A**) Relative to the asthenosphere, the shallow lower mantle is enhanced by basalt (see translucent bars). (**B**) Model snapshot of composition (see fig. S6 for time series).

Our global-scale geodynamic models demonstrate that such accumulation of basalt can indeed occur within the convecting mantle (see “Global-scale geodynamic models”). The models predict an average basalt fraction of ~7.6% in the asthenospheric MORB source [that is, pyrolite by definition (*1*, *2*)] and ~15.5% in the uppermost lower mantle (Fig. 4), a difference that is consistent with our estimate for *X*_LM_ ≈ 8%. They also predict an MTZ that is strongly enhanced by basalt, consistent with one-dimensional profiles of seismic velocities (*29*, *30*). This enhancement in the models is sustained by segregation (or “unmixing”) of basalt from surrounding rock, primarily due to the breakdown of basaltic plumes near the 660. Plume breakdown at similar depths is predicted by regional-scale geodynamic models of the Hawaiian plume and consistent with seismic tomography (*31*–*33*). Basaltic material from the base of the MTZ enters the lower mantle [as an avalanche (*34*) or passively entrained] and accumulates there. Alternatively, small-scale convection within stagnant slabs may separate dense basalt from buoyant harzburgite and contribute to mantle layering (*35*). We show that such rather small-scale processes of compositional segregation, often unresolved by global-scale geodynamic models, can balance convective mixing in the long term and thus affect heat and material fluxes through the mantle.

## DISCUSSION AND CONCLUSION

Our global-scale geodynamic models demonstrate that compositional mantle layering can be sustained by, and even may be a natural consequence of, thermochemical whole-mantle convection. Our suite of separate regional-scale geodynamic models predicts that slabs stagnate at ~1000-km depth in such a moderately compositionally layered mantle. To use slab flattening at this depth (as observed in Fig. 1) as evidence for moderate compositional layering, however, alternative mechanisms for stagnation need to be evaluated.

A gradual viscosity increase through the shallow lower mantle with a viscosity maximum at ~1500-km depth has been proposed as an alternative explanation for slab flattening (*36*, *37*). We tested this hypothesis in a suite of slab-sinking models with *X*_LM_ = 0%, Γ = −1 MPa/K, and a linear increase in viscosity (more than two orders of magnitude) from a depth of 660 to 1500 km. The models show that such an increase in viscosity is sufficient to decelerate slab sinking to the point that slabs flatten near horizontally to apparently stagnate in a (tomography) snapshot (fig. S1). However, the slabs indeed continue to sink slowly through the mantle, and thus a gradual increase in viscosity alone provides no explanation for slab flattening to preferentially occur at any particular depth. In contrast, seismic tomography shows that slab flattening in the shallow lower mantle (that is, for nearly all five examples) occurs consistently at depths of 800 to 1000 km (*16*). Such a clustering of stagnation depths concurs with the predictions of our models in regime II (red squares in Fig. 3D; see fig. S2) and is thus a good indication for moderate mantle compositional layering. A combination of both mechanisms may slightly reduce the excess fraction of basalt required in the lower mantle.

**Fig. 5:**
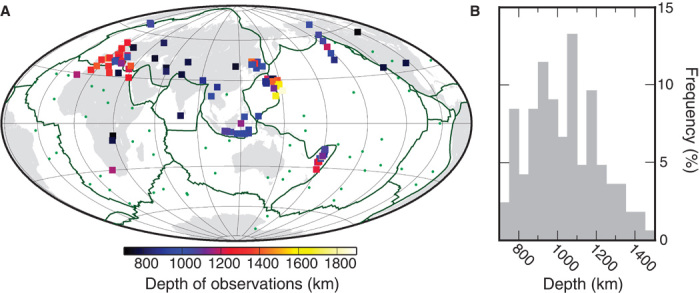
Detections of lower-mantle seismic discontinuities in the depth range of 700 to 2000 km. (**A**) Global distribution of detections shows that most detections (>75%) occur near (<1000 km) subduction zones (green lines); only ~25% occur near hot spots (green dots). (**B**) Histogram of detection depths shows a peak at depths of about 1000 to 1100 km. See table S2 for data sources.

Mantle compositional layering, in general, and slab stagnation in the uppermost lower mantle, in particular, are also consistent with numerous detections of seismic-wave conversions and reflections in the uppermost lower mantle (Fig. 5A). Without any known phase transitions in the uppermost lower mantle, wave conversions and reflections are ascribed to radial changes in mantle composition (*38*–*40*) or subhorizontal basalt layers that are less than 10 km thick (*41*). Such layers may result from basalt segregation in the MTZ or long-term slab stagnation at ~1000-km depth. Although some scatter is expected for each of these scenarios, the depths of detections indeed peak at about 1000 to 1100 km (Fig. 5B). A subset of detections near well-illuminated subduction zones confirms the presence of stagnant slabs beneath Indonesia and Tonga-Kermadec.

By integrating geodynamic modeling and seismic observations, we argue that an intrinsically dense lower mantle enriched in basalt inhibits the sinking of some slab segments beyond depths of ~1000 km. With the density of the floating slabs reasonably well defined, Archimedes’ principle allows us to infer lower-mantle density and hence composition. The resulting moderate compositional mantle layering (with a lower-mantle Mg/Si of roughly 1.18) can be sustained by segregation and gravitational settling of dense basalt. However, we do not rule out alternative (maybe primitive) dense compositions to be similarly subject to long-term segregation and concentration in the lower mantle [which may have important geochemical implications (*42*, *43*)]. Lower-mantle enhancement in Si-rich (basaltic or primitive) materials is consistent with independent cosmochemical (*10*, *11*, *27*) and geophysical estimates (*3*, *5*, *6*, *8*, *9*), although the significant uncertainties of thermodynamic and thermoelastic properties of lower-mantle rocks need to be considered (*4*, *5*, *7*). Such moderate mantle layering is further able to address geochemical differences between ocean island basalts and MORB (*44*). Quantification of lower-mantle composition can provide bounds on the ill-constrained light-element budget of the Earth’s core and, ultimately, the bulk chemistry of our planet.

## MATERIALS AND METHODS

### Methods for slab-sinking models

Using the finite-element code CITCOM, we solved the conservation equations of mass, momentum, and energy to model mantle flow (*45*). We treated the mantle as a highly viscous, infinite Prandtl number, Boussinesq fluid. Accordingly, we focused on density-driven convection, neglecting the effects of latent-heat release/consumption, as well as adiabatic and shear heating/cooling. Composition was tracked using nondiffusive tracers (*46*–*48*). We applied a second-order Runge-Kutta scheme to semi-implicitly integrate compositional advection (at the tracers) and temperature advection/diffusion (at the finite-element mesh).

To study the evolution of subducted slabs, we used a simplified two-dimensional model setup. Instead of attempting to simulate the entire complex process of subduction, we focused on modeling the free sinking of a slab segment through the mantle and the interaction of this segment with the major mantle phase transition at 660-km depth and with a compositional domain boundary initially positioned at the same depth. The modeled slab segment is initially detached from the lithosphere. Thus, we ignored any coupling between the slab and the overriding plate, as well as many other complexities related to subduction. Accordingly, our approach is similar to that of free subduction modeling (*49*–*51*).

To model slab descent, we used a rectangular model box (width, 10,000 km; depth, 2500 km) that was heated from below and cooled from above. The box’s boundaries remain closed to inflow and outflow. Side boundaries are reflective. Velocity boundary conditions at the top and bottom are no-slip and free-slip, respectively. Initially, the slab is positioned as a cool thermal anomaly centered at a distance of 5000 km from both side boundaries. The top and bottom of the slab are positioned at a distance of z* = 207.2 km and z* = 900 km from the top boundary, respectively. The initial dip angle of the slab β is varied between 30° and 60°. Here, we note that the top 400 km of the box (that is, z* < 400 km) represents a “virtual” extension of the model, which we used to “park” part of the slab. We envisioned the virtual part of the slab to represent a part that has not been subducted but is ready to subduct at the trench (again, the process of subduction itself is not explicitly modeled). Accordingly, the nonvirtual (or “real”) depth of the box is *z*_box_ = 2100 km (for governing parameters, see table S1), not 2500 km, and the real depths of the top and bottom of the slab are *z* = −192.8 km and *z* = 500 km, respectively. Note that without a virtual extension of the box, the maximum slab segment length in our model setup would be restricted to a couple of hundreds of kilometers for steep slabs.

We carefully computed the slab’s initial thermal and compositional anomaly, which ultimately act to drive slab descent (for initial conditions, see fig. S4). Slab temperatures result from the combined effects of lithospheric cooling (that is, before subduction) and slab warming (during slab descent). For example, the parts of the slab initially positioned at high *z* are somewhat warmer than those at low *z* because the former have experienced more warming during descent than the latter. Lithospheric cooling in an infinite half-space is computed analytically as a function of the age of the plate at the trench τ, which is varied between 30 and 70 My. For *z* > 0 km, the subsequent effects of slab warming due to thermal diffusion during slab descent are taken into account (that is, the deep parts of the slab segment are warmer than the shallow parts). Slab heating is assumed to have progressed with sinking time *t*_sink_ before the onset of the simulation, with *t*_sink_ = *z*/*v*_sink_. We assumed that *v*_sink_ = 60 km/My (*52*). Slab heating due to thermal diffusion along one-dimensional half-space cooling profiles with far-field temperatures of *T*_m_ is computed numerically using Fourier series expansion [see Motoki and Ballmer (*35*) for details]. Compositionally, the slab consists of a package of basalt and harzburgite. The layer of basalt is 7 km thick and positioned at the top of the slab. The underlying layer of harzburgite progressively becomes less depleted with depth (or with distance from the basalt layer). Accordingly, harzburgite gradually gives way to ambient-mantle material (that is, pyrolitic lherzolite). The specific depletion profile is derived from a separate two-dimensional numerical model of flow and melting beneath a mid-ocean ridge (*53*).

Physical and compositional changes at the upper-to-lower mantle boundary at *z* = 660 km are further key ingredients of our model. Compared to the upper mantle, we increased the initial basalt content of the lower mantle by *X*_LM_, another free parameter (see table S1, bottom four rows). Moreover, we accounted for a viscosity jump of a factor of λ = 10 at 660-km depth. Most importantly, we modeled the major effects of the endothermic phase transition in the olivine system (from a spinel structure to a perovskite structure). The transition usually occurs at 660 km but is vertically deflected in a thermally heterogeneous mantle. Because of a negative Clapeyron slope Γ, which we varied between −0.5 and −3 MPa/K (*24*), it is, for example, deflected upward in anomalously cold mantle. Because the phase transition is accompanied with a substantial density jump Δρ_660_, any such deflections directly impede mantle flow. In contrast, the effects of latent heat consumption/release at the phase transition, which we neglected (see above), are secondary (*19*, *54*). We parameterized our model such that the density of quartz-normative (that is, olivine-free) basalt remains unaffected by the phase change at 660 km.

Finally, we accounted for the most relevant depth dependencies of physical mantle properties. Here, again, we focused on the parameters that directly affect mantle density ρ and thus mantle flow. With ρ = ρ_m_ + α(*T*_m_ − *T*) + Δρ_*F*_*F* + Δρ_*X*_*X* (with *F* being depletion in peridotite and *X* being basalt content), these parameters are thermal expansivity α and the density anomalies related to basalt Δρ_*X*_ and depletion in peridotite Δρ_*F*_. Thermal expansivity α is fixed at 3 × 10^−5^ K in the upper mantle but is linearly decreased from 2.5 × 10^−5^ K^−1^ (at *z* = 660 km) to 1.4 × 10^−5^ K^−1^ (at *z* = 2100 km) in the lower mantle, a linear decrease with depth that is consistent with the work of Tosi *et al.* (*55*) (that is, for a cool adiabat). Further, we adopted a Δρ_*F*_ of −165 kg/m^3^ for *z* ≤ 300 km, a Δρ_*F*_ of −230 kg/m^3^ for 300 km < *z* ≤ 410 km, a Δρ_*F*_ of −100 kg/m^3^ for 410 km < *z* ≤ 660 km, a Δρ_*F*_ of +320 kg/m^3^ for 660 km < *z* ≤ 720 km, and a Δρ_*F*_ of −115 kg/m^3^ for *z* > 720 km, consistent with the study by Xu *et al.* (*22*). Δρ_*X*_ is generally set to Δρ_*X*_ = −1.1Δρ_*F*_. Although uncertainties remain as to the absolute value of Δρ_*X*_ in the lower mantle (*5*), we chose not to explicitly explore this parameter because of an expected direct trade-off with the model parameter *X*_LM_.

In terms of rheology, we focused on the temperature dependency of viscosity by applying an activation volume of zero and a finite activation energy E*. Viscosity is a linear function of stress (that is, Newtonian rheology), but E* is adjusted [from ~360 kJ/mol (*56*, *57*) to 180 kJ/mol] to mimic the effects of deformation by dislocation creep in addition to diffusion creep (*58*). Finally, we imposed a viscosity jump λ of a factor of 10 at 660-km depth. We ignored the effects of metastability of olivine and pyroxene (*25*, *59*) on slab behavior because they are limited to depths <660 km, or at least <740 km, and thus circumstantial for slab stagnation in the uppermost lower mantle.

### Sinking behavior of slab segments

Our numerical models are designed to investigate the sinking behavior of slabs as a function of slab and mantle parameters. Together with slab age τ and slab angle β, mantle parameters *X*_LM_ and Γ define whether a slab sinks deep into the deep mantle or at which depth it stagnates. We observed three main regimes: deep slab sinking (regime I), slab stagnation at ~1000-km depth (regime II), and slab stagnation at ~660 km depth (regime III; see the main text and fig. S3).

In all our models, the slab segments freely sink through the upper mantle, similar to “free subduction” in analog or numerical modeling (*49*–*51*). The predictions of free-subduction models in terms of subduction and trench velocities as well as τ and β can adequately reproduce global trends (*52*, *60*), showing that the buoyancy forces related to the thermal anomaly of the slab dominate the sinking of subducted slabs and surrounding mantle flow. For free subduction, the model trench (that is, in our model, the intersection of the top surface of the basalt layer with a horizontal line at *z* = 0; see fig. S4) always retreats relative to the mantle as the slab sinks. Trench retreat is indeed more common on Earth than trench advance (*60*). For the subset of sinking slabs with advancing or stationary trenches, our models are accordingly not generally applicable. These slabs commonly buckle and/or pile up in the MTZ or uppermost lower mantle, often near 1000-km depth (*16*, *61*–*65*), and episodically flush into the lower mantle (*66*), a behavior that is consistent with the impeding effects of a compositionally layered mantle on slab sinking.

As soon as the sinking slab segment in our models starts to punch through the 660 and dip into the underlying high-viscosity (and high-density) lower mantle, the slab’s tip is sharply decelerated. Consequently, the slab segment as a whole rotates counterclockwise. In a subset of cases, this rotation critically changes the inclination of the slab’s tail such that the tail does not penetrate the 660 (penetration is favored for high β, see Fig. 3D), although the slab’s tip already entered the lower mantle. For these cases, the tip and tail of the slab ultimately stagnate just below and just above the 660, respectively. This scenario represents a hybrid regime between regimes II and III (cf. red-and-green bicolored squares in Fig. 3D). However, the occurrence of this hybrid regime remains, to some extent, an artifact of our model setup, because the finite length of slab segments favors rotation. Nevertheless, natural slab dip angles in the MTZ may vary along the trench (for example, because of trench curvature), allowing one flank of the slab but not the other flank to cross the 660. Such a behavior can explain along-arc variations in the depth of slab stagnation, as are found to be common for circum-Pacific subduction zones (for example, Tonga-Kermadec and Central America) (*16*).

In a very narrow parameter range (*X*_LM_ = 10% and τ = 70 My), we further found another hybrid regime between regimes I and II. In this regime (cf. red-and-blue bicolored squares in Fig. 3D), the slabs sink to depths >1200 km but lack the negative thermal buoyancy to sink >1600 km before they are conductively heated to slowly rebound upward. This behavior is explained by the depth dependency of thermal expansivity in the lower mantle (*55*) (cf. Fig. 2A), which causes slab density to decrease with increasing depth. Depth dependency of thermal expansivity thus acts to fine-tune the level of neutral buoyancy for the slabs in this hybrid regime. The rare occurrence of this hybrid regime does not compromise our conclusion that stagnation depths of ~660 and ~1000 km (fig. S3), along with deep slab sinking, are the three dominant regimes of slab behavior in a compositionally stratified mantle.

### Global-scale geodynamic models

To test whether mantle layering can be sustained within the convecting mantle, we explored global-scale thermochemical mantle-convection models. We used the finite-volume code STAGYY to solve these models in a spherical annulus geometry (*67*, *68*). Mantle composition is modeled as a mechanical mixture of two end members: harzburgite and MORB [for details, see Nakagawa and Tackley (*69*)].

We solved three different cases (A1, A2, and B1) with boundary conditions and model setup adopted from the study by Nakagawa and Tackley (*69*), but with a higher model resolution (cases A1 and B1). Except for the surface yield stress parameterization, all parameters were taken from Nakagawa and Tackley (*69*). We adopted an updated yield-stress parameterization [from Crameri and Tackley (*70*)] to simulate plate-like behavior with more realistic plate sizes and crustal thicknesses. The density difference between basalt and harzburgite is a function of depth in the upper mantle (*69*). In the lower mantle, it is assumed to be constant (1.35%) for cases A1 and A2 and to decrease linearly with depth [from 1.35% at the 660 to 0.75% at the core-mantle boundary (CMB)] for case B1. The high-resolution cases A1 and B1 (2048 × 256 grid points) are shown in Fig. 4 and fig. S5, respectively. The lower-resolution case A2 (1024 × 128 grid points) is analogous to the case shown in Fig. 4B (right panel) in Nakagawa and Tackley (*69*).

The models predict that the Earth’s mantle is a mechanical mixture (“marble cake”) of basaltic and harzburgitic materials (*22*, *71*, *72*). Close to the surface, mantle materials melt to form a crust composed of basalt underlain by a harzburgitic residue. At subduction zones, this compositional heterogeneity enters the mantle and is stirred by convection. Lateral compositional heterogeneity is pronounced and strongly time-dependent (figs. S5 and S6). Nevertheless, average composition varies systematically with depth, as basalt and harzburgite are subject to different driving forces due to distinct radial density profiles.

Our models predict that basaltic material becomes strongly concentrated in the MTZ (*28*). This concentration occurs as the buoyancy of basalt (that is, density anomaly relative to the ambient mantle) is strongly negative above and positive below the MTZ (see arrows in Fig. 2B). In the models, the dominant process for basalt concentration in the MTZ is the breakdown of plumes that carry basalt from the lower mantle across the 660. During this process, segregation of the plume’s compounds occurs: basalt tends to settle at the bottom of the MTZ, whereas harzburgite tends to rise further into the asthenosphere. Segregation is driven by the density difference between (buoyant) harzburgite and (dense) basalt in the MTZ and facilitated by the relatively low viscosities above the 660 [that is, a factor of 30 smaller than below (*69*)].

Over long time scales, basaltic materials are further predicted to successively accumulate in the lower mantle. Accumulation in the lower mantle is expected to occur because basaltic materials are negatively buoyant throughout the mantle except for a narrow depth range between 660 and ~750 km (*23*). This depth range of density reversal between mafic and ultramafic lithologies acts as a “basalt barrier,” at first inhibiting the flow of basalt into the lower mantle (*73*). However, basaltic material that accumulated at the base of the MTZ crosses the narrow basalt barrier as it is entrained by downward flow (for example, by a subducted slab) or periodically collapses under its own weight as an avalanche, similar to those predicted by Tackley *et al.* (*34*). In addition, subducted slabs can directly carry relatively “fresh” MORB into the lower mantle. Dense basalt in the lower mantle not only is predicted to accumulate as piles near the CMB, which is a possible explanation for the large low shear-wave velocity provinces (LLSVPs) (*74*–*78*), but also remains entrained to increase the basalt fraction throughout the lower mantle (Fig. 4A).

The amount of basalt remaining entrained versus settling near the CMB depends on the density profile of basalt in the lower mantle. In cases A1 and A2 with a density difference of 1.35% between basalt and harzburgite at the CMB, more basalt settles near the CMB (and less basalt remains entrained) than in case B1 with a density difference of 0.75% (fig. S5B versus fig. S6B). However, the relative enhancement of the shallow lower mantle by basalt (relative to the asthenospheric MORB source, that is, pyrolite by definition) remains similar in all cases (about 8 to 10%). Nevertheless, cases A1 and A2, and B1 have different implications for bulk silicate Earth compositions, with the latter (case B1) remaining more realistic, because the former (cases A1 and A2) overpredict LLSVP volumes. Moreover, in addition to basalt accumulation at the CMB, primitive material may contribute to LLSVP compositions and volumes (*69*, *78*–*80*).

Except for the dense piles at the CMB, which successively grow over time, the compositional layering of the mantle is near complete at ~1 Gy with only minor changes thereafter. After ~1 Gy, segregation of basalt from harzburgite and mechanical mixing are predicted to be well balanced. Figure S6A shows that the general compositional layering with harzburgite enhancement in the asthenosphere, as well as basalt enhancement in the lower mantle and MTZ, is independent of model resolution. This layering can account for the long-term geochemical enrichment and depletion of the ocean island basalt and MORB sources, respectively, as is constrained by isotope geochemistry (*44*).

Subducted slabs in the global-scale models interact with mantle compositional layering. Consistent with regional-scale models (Fig. 3), global-scale models predict slab flattening to occur above and below the 660 (snapshots in figs. S5 and S6). Slabs commonly flatten in the uppermost lower mantle but are often subsequently (albeit very slowly) pushed deeper into the mantle as slab subduction continues to inject material from above. The rate at which flattened slabs sink through the lower mantle is thus sensitive to the locations and migration rates of the trenches above. Because the slab equilibrates thermally as this process continues, subhorizontal slab-like thermal anomalies are predicted to be restricted to the shallow lower mantle (and MTZ). Thermal equilibration may accordingly render the slab invisible to seismic tomography. In addition, convective instability rising out of the slab’s underbelly may disintegrate stagnant slabs to promote seismic invisibility (*35*). The observation from seismic tomography that flattened slab segments, in the MTZ and at ~1000-km depth, display a finite horizontal length indeed suggests a mechanism for disintegration of stagnant slabs.

## Supplementary Material

http://advances.sciencemag.org/cgi/content/full/1/11/e1500815/DC1

## SUPPLEMENTARY MATERIALS

Supplementary material for this article is available at http://advances.sciencemag.org/cgi/content/full/1/11/e1500815/DC1 Fig. S1. Numerical-model predictions of slab descent through a mantle with a gradual increase in viscosity between 660- and 1500-km depths. Fig. S2. Histogram of predicted stagnation depths for slabs that stagnate in the uppermost lower mantle. Fig. S3. Numerical-model predictions of slab descent as a time series. Fig. S4. Initial condition of the center of the numerical-model box for a case with τ = 50 My, β = 45°, and *X*_LM_ = 10%. Fig. S5. Compositional mantle evolution predicted by global-scale geodynamic models for different lower-mantle density profiles of basalt. Fig. S6. Compositional mantle evolution predicted by global-scale geodynamic models. Table S1. Notations. Table S2. Sources of data for Fig. 5. Table S3. Hypothetical (molar) abundances of major oxides in the lower mantle. References (*81*–*96*)

## References

[1] A. E. Ringwood, *Origin of the Earth and Moon* (Springer, Berlin, Germany, 1979).

[2] Workman R. K., Hart S. R., Major and trace element composition of the depleted MORB mantle (DMM). Earth Planet. Sci. Lett. 231, 53–72 (2005).

[3] Murakami M., Ohishi Y., Hirao N., Hirose K., A perovskitic lower mantle inferred from high-pressure, high-temperature sound velocity data. Nature 485, 90–94 (2012). 22552097 10.1038/nature11004

[4] Cottaar S., Heister T., Rose I., Unterborn C., BurnMan: A lower mantle mineral physics toolkit. Geochem. Geophys. Geosyst. 15, 1164–1179 (2014).

[5] Cobden L., Goes S., Ravenna M., Styles E., Cammarano F., Gallagher K., Connolly J. A. D., Thermochemical interpretation of 1-D seismic data for the lower mantle: The significance of nonadiabatic thermal gradients and compositional heterogeneity. J. Geophys. Res. 114, B11309 (2009).

[6] Matas J., Bass J., Ricard Y., Mattern E., Bukowinski M. S. T., On the bulk composition of the lower mantle: Predictions and limitations from generalized inversion of radial seismic profiles. Geophys. J. Int. 170, 764–780 (2007).

[7] Mattern E., Matas J., Ricard Y., Bass J., Lower mantle composition and temperature from mineral physics and thermodynamic modelling. Geophys. J. Int. 160, 973–990 (2005).

[8] Khan A., Connolly J. A. D., Taylor S. R., Inversion of seismic and geodetic data for the major element chemistry and temperature of the Earth’s mantle. J. Geophys. Res. Solid Earth 113, B09308 (2008).

[9] Ricolleau A., Fei Y., Cottrell E., Watson H., Deng L., Zhang L., Fiquet G., Auzende A.-L., Roskosz M., Morard G., Prakapenka V., Density profile of pyrolite under the lower mantle conditions. Geophys. Res. Lett. 36, L06302 (2009).

[10] Javoy M., The integral enstatite chondrite model of the earth. Geophys. Res. Lett. 22, 2219–2222 (1995).

[11] McDonough W. F., Sun S.-s., The composition of the Earth. Chem. Geol. 120, 223–253 (1995).

[12] Allègre C. J., Poirier J.-P., Humler E., Hofmann A. W., The chemical composition of the Earth. Earth Planet. Sci. Lett. 134, 515–526 (1995).

[13] Grand S. P., van der Hilst R. D., Widiyantoro S., Global seismic tomography: A snapshot of convection in the Earth. GSA Today 7, 1–7 (1997).11541665

[14] van der Meer D. G., Spakman W., van Hinsbergen D. J. J., Amaru M. L., Torsvik T. H., Towards absolute plate motions constrained by lower-mantle slab remnants. Nat. Geosci. 3, 36–40 (2010).

[15] Ritsema J., Deuss A., van Heijst H. J., Woodhouse J. H., S40RTS: A degree-40 shear-velocity model for the mantle from new Rayleigh wave dispersion, teleseismic traveltime and normal-mode splitting function measurements. Geophys. J. Int. 184, 1223–1236 (2011).

[16] Fukao Y., Obayashi M., Subducted slabs stagnant above, penetrating through, and trapped below the 660 km discontinuity. J. J. Geophys. Res. Solid Earth 118, 5920–5938 (2013).

[17] van der Hilst R. D., Widiyantoro S., Engdahl E. R., Evidence for deep mantle circulation from global tomography. Nature 386, 578–584 (1997).

[18] van Keken P., Zhong S., Mixing in a 3D spherical model of present-day mantle convection. Earth Planet. Sci. Lett. 171, 533–547 (1999).

[19] Christensen U. R., Yuen D. A., The interaction of a subducting lithospheric slab with a chemical or phase boundary. J. Geophys. Res. 89, 4389–4402 (1984).

[20] Billen M. I., Slab dynamics in the transition zone. Phys. Earth Planet. Inter. 183, 296–308 (2010).

[21] King S. D., Frost D. J., Rubie D. C., Why cold slabs stagnate in the transition zone. Geology 43, 231–234 (2015).

[22] Xu W., Lithgow-Bertelloni C., Stixrude L., Ritsema J., The effect of bulk composition and temperature on mantle seismic structure. Earth Planet. Sci. Lett. 275, 70–79 (2008).

[23] Hirose K., Fei Y., Ma Y., Mao H.-K., The fate of subducted basaltic crust in the Earth’s lower mantle. Nature 397, 53–56 (1999).

[24] Ghosh S., Ohtani E., Litasov K. D., Suzuki A., Dobson D., Funakoshi K., Effect of water in depleted mantle on post-spinel transition and implication for 660 km seismic discontinuity. Earth Planet. Sci. Lett. 371–372, 103–111 (2013).

[25] Karato S.-i., Riedel M. R., Yuen D. A., Rheological structure and deformation of subducted slabs in the mantle transition zone: Implications for mantle circulation and deep earthquakes. Phys. Earth Planet. Inter. 127, 83–108 (2001).

[26] Elkins-Tanton L. T., Linked magma ocean solidification and atmospheric growth for Earth and Mars. Earth Planet. Sci. Lett. 271, 181–191 (2008).

[27] Kaminski E., Javoy M., A two-stage scenario for the formation of the Earth’s mantle and core. Earth Planet. Sci. Lett. 365, 97–107 (2013).

[28] Anderson D. L., Bass J. D., Transition region of the Earth’s upper mantle. Nature 320, 321–328 (1986).

[29] Cammarano F., Romanowicz B., Insights into the nature of the transition zone from physically constrained inversion of long-period seismic data. Proc. Natl. Acad. Sci. U.S.A. 104, 9139–9144 (2007). 17483461 10.1073/pnas.0608075104 PMC1890460

[30] Cammarano F., Romanowicz B., Stixrude L., Lithgow-Bertelloni C., Xu W., Inferring the thermochemical structure of the upper mantle from seismic data. Geophys. J. Int. 179, 1169–1185 (2009).

[31] Ballmer M. D., Ito G., Wolfe C. J., Solomon S. C., Double layering of a thermochemical plume in the upper mantle beneath Hawaii. Earth Planet. Sci. Lett. 376, 155–164 (2013).

[32] C. Cheng, R. M. Allen, R. W. Porritt, M. D. Ballmer, Seismic constraints on a double-layered asymmetric whole-mantle plume beneath Hawaii, in *Hawaiian Volcanism, From Source to Surface*, R. Carey, M. Poland, V. Cayol, D. Weis, Eds. (John Wiley & Sons Inc., Hoboken, NJ, 2015), pp. 19–34.

[33] M. D. Ballmer, G. Ito, C. Cheng, Asymmetric dynamical behavior of thermochemical plumes and implications for Hawaiian lava composition, in *Hawaiian Volcanism, From Source to Surface*, R. Carey, M. Poland, V. Cayol, D. Weis, Eds. (John Wiley & Sons Inc., Hoboken, NJ, 2015), pp. 36–57.

[34] Tackley P. J., Stevenson D. J., Glatzmaier G. A., Schubert G., Effects of an endothermic phase transition at 670 km depth in a spherical model of convection in the Earth’s mantle. Nature 361, 699–704 (1993).

[35] Motoki M. H., Ballmer M. D., Intraplate volcanism due to convective instability of stagnant slabs in the Mantle Transition Zone. Geochem. Geophys. Geosyst. 16, 538–551 (2015).

[36] Marquardt H., Miyagi L., Slab stagnation in the shallow lower mantle linked to an increase in mantle viscosity. Nat. Geosci. 8, 311–314 (2015).

[37] Morra G., Yuen D. A., Boschi L., Chatelain P., Koumoutsakos P., Tackley P. J., The fate of the slabs interacting with a density/viscosity hill in the mid-mantle. Phys. Earth Planet. Inter. 180, 271–282 (2010).

[38] Kawakatsu H., Niu F., Seismic evidence for a 920-km discontinuity in the mantle. Nature 371, 301–305 (1994).

[39] Vinnik L. P., Oreshin S. I., Speziale S., Weber M., Mid-mantle layering from SKS receiver functions. Geophys. Res. Lett. 37, L24302 (2010).

[40] Vanacore E., Niu F., Kawakatsu H., Observations of the mid-mantle discontinuity beneath Indonesia from S to P converted waveforms. Geophys. Res. Lett. 33, L04302 (2006).

[41] Niu F., Kawakatsu H., Fukao Y., Seismic evidence for a chemical heterogeneity in the midmantle: A strong and slightly dipping seismic reflector beneath the Mariana subduction zone. J. Geophys. Res. Solid Earth 108, 2419 (2003).

[42] Kellogg L. H., Hager B. H., van der Hilst R. D., Compositional stratification in the deep mantle. Science 283, 1881–1884 (1999). 10082454 10.1126/science.283.5409.1881

[43] Becker T. W., Kellogg J. B., O’Connell R. J., Thermal constraints on the survival of primitive blobs in the lower mantle. Earth Planet. Sci. Lett. 171, 351–365 (1999).

[44] Hofmann A. W., Mantle geochemistry: The message from oceanic volcanism. Nature 385, 219–229 (1997).

[45] Moresi L., Zhong S., Gurnis M., The accuracy of finite element solutions of Stokes’s flow with strongly varying viscosity. Phys. Earth Planet. Inter. 97, 83–94 (1996).

[46] Gerya T. V., Yuen D. A., Characteristics-based marker-in-cell method with conservative finite-differences schemes for modeling geological flows with strongly variable transport properties. Phys. Earth Planet. Inter. 140, 293–318 (2003).

[47] van Keken P. E., King S. D., Schmeling H., Christensen U. R., Neumeister D., Doin M.-P., A comparison of methods for the modeling of thermochemical convection. J. Geophys. Res. 102, 22477–22495 (1997).

[48] Tackley P. J., King S. D., Testing the tracer ratio method for modeling active compositional fields in mantle convection simulations. Geochem. Geophys. Geosyst. 4, 8302 (2003).

[49] Schellart W. P., Kinematics of subduction and subduction-induced flow in the upper mantle. J. Geophys. Res. Solid Earth 109, B07401 (2004).

[50] Bellahsen N., Faccenna C., Funiciello F., Dynamics of subduction and plate motion in laboratory experiments: Insights into the “plate tectonics” behavior of the Earth. J. Geophys. Res. Solid Earth 110, B01401 (2005).

[51] Capitanio F. A., Morra G., Goes S., Dynamic models of downgoing plate-buoyancy driven subduction: Subduction motions and energy dissipation. Earth Planet. Sci. Lett. 262, 284–297 (2007).

[52] Goes S., Capitanio F. A., Morra G., Seton M., Giardini D., Signatures of downgoing plate-buoyancy driven subduction in Cenozoic plate motions. Phys. Earth Planet. Inter. 184, 1–13 (2011).

[53] Ballmer M. D., van Hunen J., Ito G., Bianco T. A., Tackley P. J., Intraplate volcanism with complex age-distance patterns: A case for small-scale sublithospheric convection. Geochem. Geophys. Geosyst. 10, Q06015 (2009).

[54] Christensen U. R., Yuen D. A., Layered convection induced by phase transitions. J. Geophys. Res. 90, 10291–10300 (1985).

[55] Tosi N., Yuen D. A., de Koker N., Wentzcovitch R. M., Mantle dynamics with pressure- and temperature-dependent thermal expansivity and conductivity. Phys. Earth Planet. Inter. 217, 48–58 (2013).

[56] Karato S.-i., Wu P., Rheology of the upper mantle: A synthesis. Science 260, 771–778 (1993). 17746109 10.1126/science.260.5109.771

[57] Hirth G., Laboratory constraints on the rheology of the upper mantle. Rev. Mineral. Geochem. 51, 97–120 (2002).

[58] Christensen U., Convection with pressure- and temperature-dependent non-Newtonian rheology. Geophys. J. Int. 77, 343–384 (1984).

[59] Agrusta R., van Hunen J., Goes S., The effect of metastable pyroxene on the slab dynamics. Geophys. Res. Lett. 41, 8800–8808 (2014).

[60] Schellart W. P., Stegman D. R., Freeman J., Global trench migration velocities and slab migration induced upper mantle volume fluxes: Constraints to find an Earth reference frame based on minimizing viscous dissipation. Earth-Sci. Rev. 88, 118–144 (2008).

[61] Capitanio F. A., Stegman D. R., Moresi L. N., Sharples W., Upper plate controls on deep subduction, trench migrations and deformations at convergent margins. Tectonophysics 483, 80–92 (2010).

[62] Ribe N. M., Stutzmann E., Ren Y., van der Hilst R., Buckling instabilities of subducted lithosphere beneath the transition zone. Earth Planet. Sci. Lett. 254, 173–179 (2007).

[63] Fukao Y., Widiyantoro S., Obayashi M., Stagnant slabs in the upper and lower mantle transition region. Rev. Geophys. 39, 291–323 (2001).

[64] Lee C., King S. D., Dynamic buckling of subducting slabs reconciles geological and geophysical observations. Earth Planet. Sci. Lett. 312, 360–370 (2011).

[65] Ribe N. M., Bending mechanics and mode selection in free subduction: A thin-sheet analysis. Geophys. J. Int. 180, 559–576 (2010).

[66] Goes S., Capitanio F. A., Morra G., Evidence of lower-mantle slab penetration phases in plate motions. Nature 451, 981–984 (2008). 18288192 10.1038/nature06691

[67] Tackley P. J., Modelling compressible mantle convection with large viscosity contrasts in a three-dimensional spherical shell using the yin-yang grid. Phys. Earth Planet. Inter. 171, 7–18 (2008).

[68] Hernlund J. W., Tackley P. J., Modeling mantle convection in the spherical annulus. Phys. Earth Planet. Inter. 171, 48–54 (2008).

[69] Nakagawa T., Tackley P. J., Influence of combined primordial layering and recycled MORB on the coupled thermal evolution of Earth’s mantle and core. Geochem. Geophys. Geosyst. 15, 619–633 (2014).

[70] Crameri F., Tackley P. J., Spontaneous development of arcuate single-sided subduction in global 3-D mantle convection models with a free surface. J. Geophys. Res. Solid Earth 119, 5921–5942 (2014).

[71] Morgan J. P., Morgan W. J., Two-stage melting and the geochemical evolution of the mantle: A recipe for mantle plum-pudding. Earth Planet. Sci. Lett. 170, 215–239 (1999).

[72] Hofmann A. W., Chemical differentiation of the Earth: The relationship between mantle, continental crust, and oceanic crust. Earth Planet. Sci. Lett. 90, 297–314 (1988).

[73] Nakagawa T., Buffett B. A., Mass transport mechanism between the upper and lower mantle in numerical simulations of thermochemical mantle convection with multicomponent phase changes. Earth Planet. Sci. Lett. 230, 11–27 (2005).

[74] McNamara A. K., Zhong S., Thermochemical structures beneath Africa and the Pacific Ocean. Nature 437, 1136–1139 (2005). 16237440 10.1038/nature04066

[75] Davaille A., Simultaneous generation of hotspots and superswells by convection in a heterogeneous planetary mantle. Nature 402, 756–760 (1999).

[76] Tan E., Gurnis M., Compressible thermochemical convection and application to lower mantle structures. J. Geophys. Res. Solid Earth 112, B06304 (2007).

[77] Garnero E. J., McNamara A. K., Structure and dynamics of Earth’s lower mantle. Science 320, 626–628 (2008). 18451293 10.1126/science.1148028

[78] Tackley P. J., Dynamics and evolution of the deep mantle resulting from thermal, chemical, phase and melting effects. Earth-Sci. Rev. 110, 1–25 (2012).

[79] Li M., McNamara A. K., Garnero E. J., Chemical complexity of hotspots caused by cycling oceanic crust through mantle reservoirs. Nat. Geosci. 7, 366–370 (2014).

[80] Deschamps F., Cobden L., Tackley P. J., The primitive nature of large low shear-wave velocity provinces. Earth Planet. Sci. Lett. 349–350, 198–208 (2012).

[81] An Y., Gu Y. J., Sacchi M. D., Imaging mantle discontinuities using least squares Radon transform. J. Geophys. Res. Solid Earth 112, B10303 (2007).

[82] Deuss A., Global observations of mantle discontinuities using SS and PP precursors. Surv. Geophys. 30, 301–326 (2009).

[83] Deuss A., Woodhouse J. H., A systematic search for mantle discontinuities using SS-precursors. Geophys. Res. Lett. 29, 90.1–90.4 (2002).

[84] Andrews J., Deuss A., Detailed nature of the 660 km region of the mantle from global receiver function data. J. Geophys. Res. Solid Earth 113, B06304 (2008).

[85] Shen Y., Wolfe C. J., Solomon S. C., Seismological evidence for a mid-mantle discontinuity beneath Hawaii and Iceland. Earth Planet. Sci. Lett. 214, 143–151 (2003).

[86] van der Meijde M., van der Lee S., Giardini D., Seismic discontinuities in the Mediterranean mantle. Phys. Earth Planet. Inter. 148, 233–250 (2005).

[87] Castle J. C., Creager K. C., A steeply dipping discontinuity in the lower mantle beneath Izu-Bonin. J. Geophys. Res. Solid Earth 104, 7279–7292 (1999).

[88] Niu F., Distinct compositional thin layers at mid-mantle depths beneath northeast China revealed by the USArray. Earth Planet. Sci. Lett. 402, 305–312 (2014).

[89] Kaneshima S., Helffrich G., Detection of lower mantle scatterers northeast of the Marianna subduction zone using short-period array data. J. Geophys. Res. Solid Earth 103, 4825–4838 (1998).

[90] Kaneshima S., Helffrich G., Dipping low-velocity layer in the mid-lower mantle: Evidence for geochemical heterogeneity. Science 283, 1888–1892 (1999). 10082456 10.1126/science.283.5409.1888

[91] Li J., Yuen D. A., Mid-mantle heterogeneities associated with Izanagi plate: Implications for regional mantle viscosity. Earth Planet. Sci. Lett. 385, 137–144 (2014).

[92] Niu F., Kawakatsu H., Depth variation of the mid-mantle seismic discontinuity. Geophys. Res. Lett. 24, 429–432 (1997).

[93] Vinnik L., Kato M., Kawakatsu H., Search for seismic discontinuities in the lower mantle. Geophys. J. Int. 147, 41–56 (2001).

[94] Vinnik L., Niu F., Kawakatsu H., Broadband converted phases from midmantle discontinuities. Earth Planets Space 50, 987–997 (1998).

[95] Day E. A., Deuss A., Reconciling *PP* and *P’P’* precursor observations of a complex 660 km seismic discontinuity. Geophys. J. Int. 194, 834–838 (2013).

[96] LeStunff Y., Wicks C. W. Jr, Romanowicz B., P′P′ precursors under Africa: Evidence for mid-mantle reflectors. Science 270, 74–77 (1995).

