# Supplementary material for: Compositional mantle layering revealed by slab stagnation at ~1000-km depth
Source: Sci Adv. 2015 Dec 10;1(11):e1500815. doi: 10.1126/sciadv.1500815 (PMC4730845; doi:10.1126/sciadv.1500815)
Supplement: http://advances.sciencemag.org/cgi/content/full/1/11/e1500815/DC1 [file supp_1_11_e1500815__index.html]

Science Advances | Science Advances

## Supplementary Materials

**This PDF file includes:**

- Fig. S1. Numerical-model predictions of slab descent through a mantle with a gradual increase in viscosity between 660- and 1500-km depths.
- Fig. S2. Histogram of predicted stagnation depths for slabs that stagnate in the uppermost lower mantle.
- Fig. S3. Numerical-model predictions of slab descent as a time series.
- Fig. S4. Initial condition of the center of the numerical-model box for a case with τ = 50 My, β = 45°, and *X*LM = 10%.
- Fig. S5. Compositional mantle evolution predicted by global-scale geodynamic models for different lower-mantle density profiles of basalt.
- Fig. S6. Compositional mantle evolution predicted by global-scale geodynamic models.
- Table S1. Notations.
- Table S2. Sources of data for Fig. 5.
- Table S3. Hypothetical (molar) abundances of major oxides in the lower mantle.
- References (*81–96*)

Download PDF

**Files in this Data Supplement:**

- Adobe PDF - 1500815\_SM.pdf
