# Supplementary material for: Compositional mantle layering revealed by slab stagnation at ~1000-km depth
Source: Sci Adv. 2015 Dec 10;1(11):e1500815. doi: 10.1126/sciadv.1500815 (PMC4730845; doi:10.1126/sciadv.1500815)
Supplement: http://advances.sciencemag.org/cgi/content/full/1/11/e1500815/DC1 [file 1500815_SM.pdf]

Science Advances
AAAS

advances.sciencemag.org/cgi/content/full/1/11/e1500815/DC1

Supplementary Materials for

Compositional mantle layering revealed by slab stagnation at ~1000-km depth

Maxim D. Ballmer, Nicholas C. Schmerr, Takashi Nakagawa, Jeroen Ritsema

Published 10 December 2015, *Sci. Adv.* **1**, e1500815 (2015)
DOI: 10.1126/sciadv.1500815

The PDF file includes:

Fig. S1. Numerical-model predictions of slab descent through a mantle with a gradual increase in viscosity between 660- and 1500-km depths.
Fig. S2. Histogram of predicted stagnation depths for slabs that stagnate in the uppermost lower mantle.
Fig. S3. Numerical-model predictions of slab descent as a time series.
Fig. S4. Initial condition of the center of the numerical-model box for a case with τ = 50 My, β = 45°, and X_{LM} = 10%.
Fig. S5. Compositional mantle evolution predicted by global-scale geodynamic models for different lower-mantle density profiles of basalt.
Fig. S6. Compositional mantle evolution predicted by global-scale geodynamic models.
Table S1. Notations.
Table S2. Sources of data for Fig. 5.
Table S3. Hypothetical (molar) abundances of major oxides in the lower mantle.
References (*81–96*)

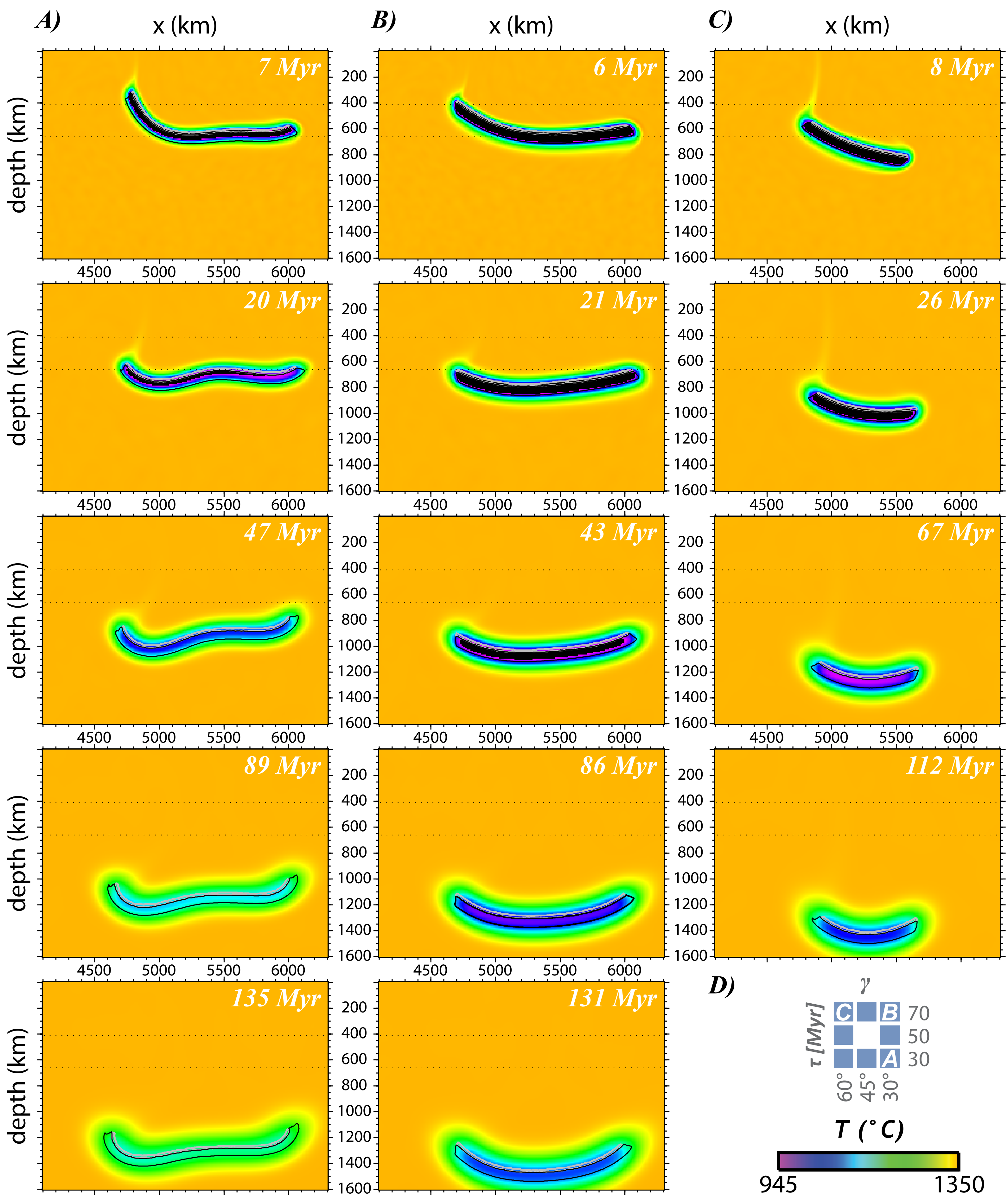

A)
B)
C)
x (km)
depth (km)
7 Myr
6 Myr
8 Myr
20 Myr
21 Myr
26 Myr
47 Myr
43 Myr
67 Myr
89 Myr
86 Myr
112 Myr
135 Myr
131 Myr
200
400
600
800
1000
1200
1400
1600
4500
5000
5500
6000
D)
γ
τ [Myr]
C
B
A
70
50
30
60°
45°
30°
T (°C)
945
1350

Fig. S1. Numerical-model predictions of slab descent through a mantle with a gradual increase in viscosity between 660- and 1500-km depths (*36*). Mantle parameters are fixed at X_{LM} = 0% and Γ = -1 MPa/K. The three columns (**A**-**C**) show time-series of three example cases, each with distinct slab parameters. Panel (**D**) provides the appropriate regime diagram, indicating the three cases shown (letters), and highlighting that all cases with a gradual increase in viscosity display deep slab-sinking behavior. For description, see Figure 3 (for visibility, arrows are removed).

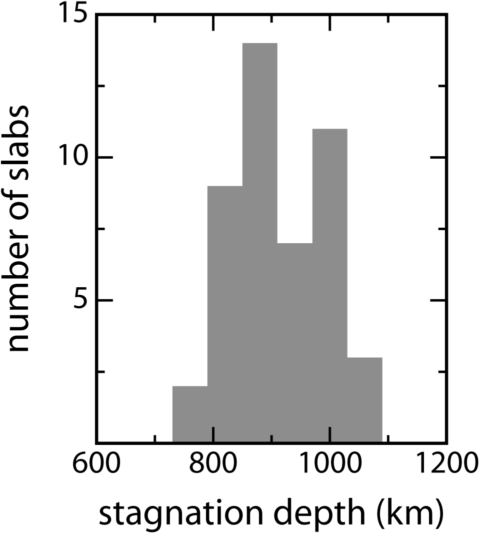

15
10
5
600
800
1000
1200
number of slabs
stagnation depth (km)

Fig. S2. Histogram of predicted stagnation depths for slabs that stagnate in the uppermost lower mantle (red squares in Fig. 3D). Stagnation depths are calculated as the minimum of averaged vertical thermal profiles. Thus, they are representative of the coolest core of the slabs (i.e., in between the slab's top and bottom). Stagnation depths are predicted to peak at ~900 km, consistent with seismic tomography images of sub-horizontal slab tops at ~800 and slab bottoms at ~1,000 km depth (*16*).

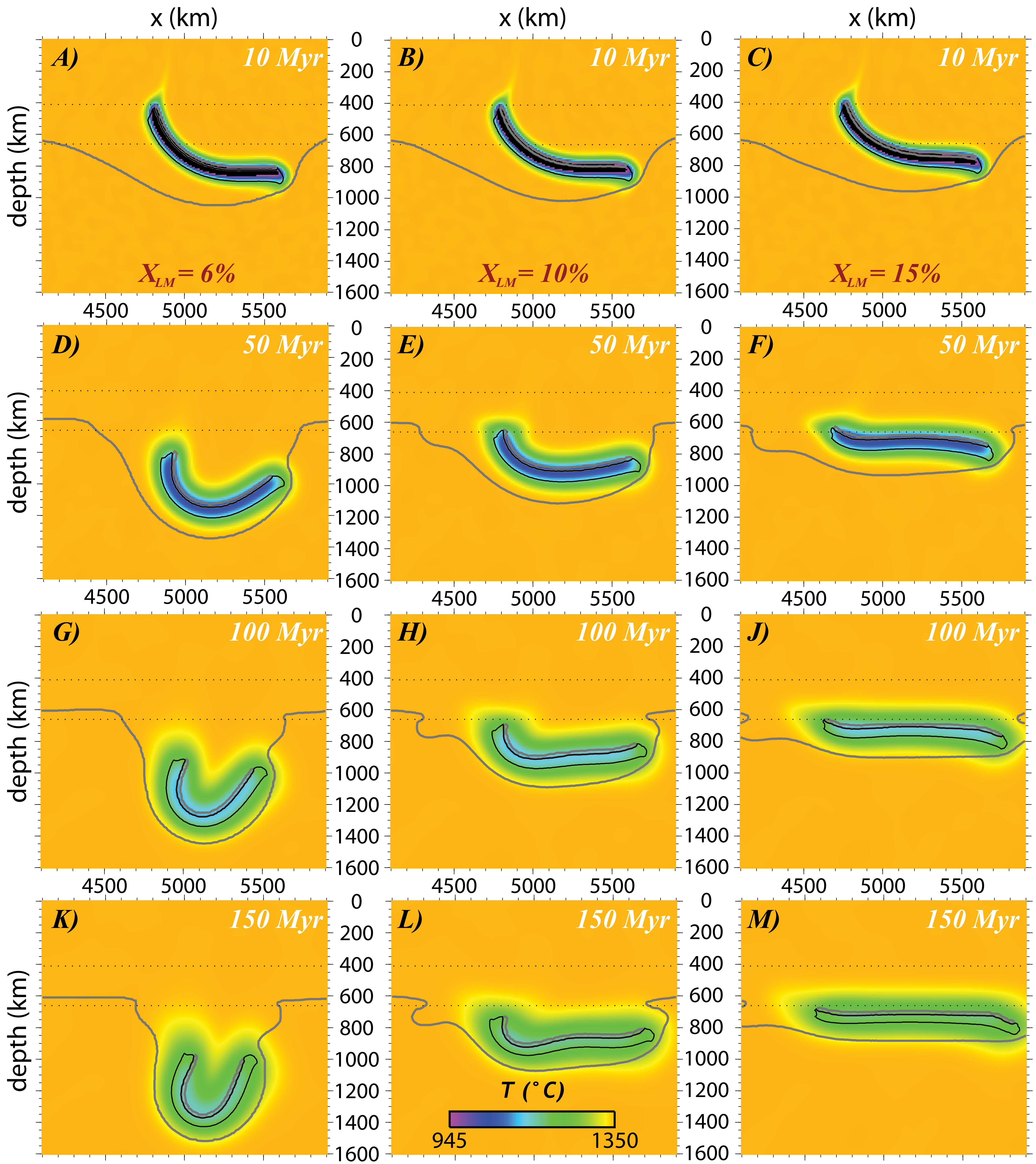

x (km)
depth (km)
A) 10 Myr
B) 10 Myr
C) 10 Myr
X_{LM}= 6%
X_{LM}= 10%
X_{LM}= 15%
D) 50 Myr
E) 50 Myr
F) 50 Myr
G) 100 Myr
H) 100 Myr
J) 100 Myr
K) 150 Myr
L) 150 Myr
M) 150 Myr
0
200
400
600
800
1000
1200
1400
1600
4500
5000
5500
T (°C)
945
1350

Fig. S3. Numerical-model predictions of slab descent as a time series with snapshots at 10, 50, 100, and 150 My (from top to bottom). The three columns are the same three cases as in Figures 3A-C (right column: X_{LM} = 6%; central column: X_{LM} = 10%; left column: X_{LM} = 15%). For description, see Figure 3 (for visibility, arrows and hatching are removed).

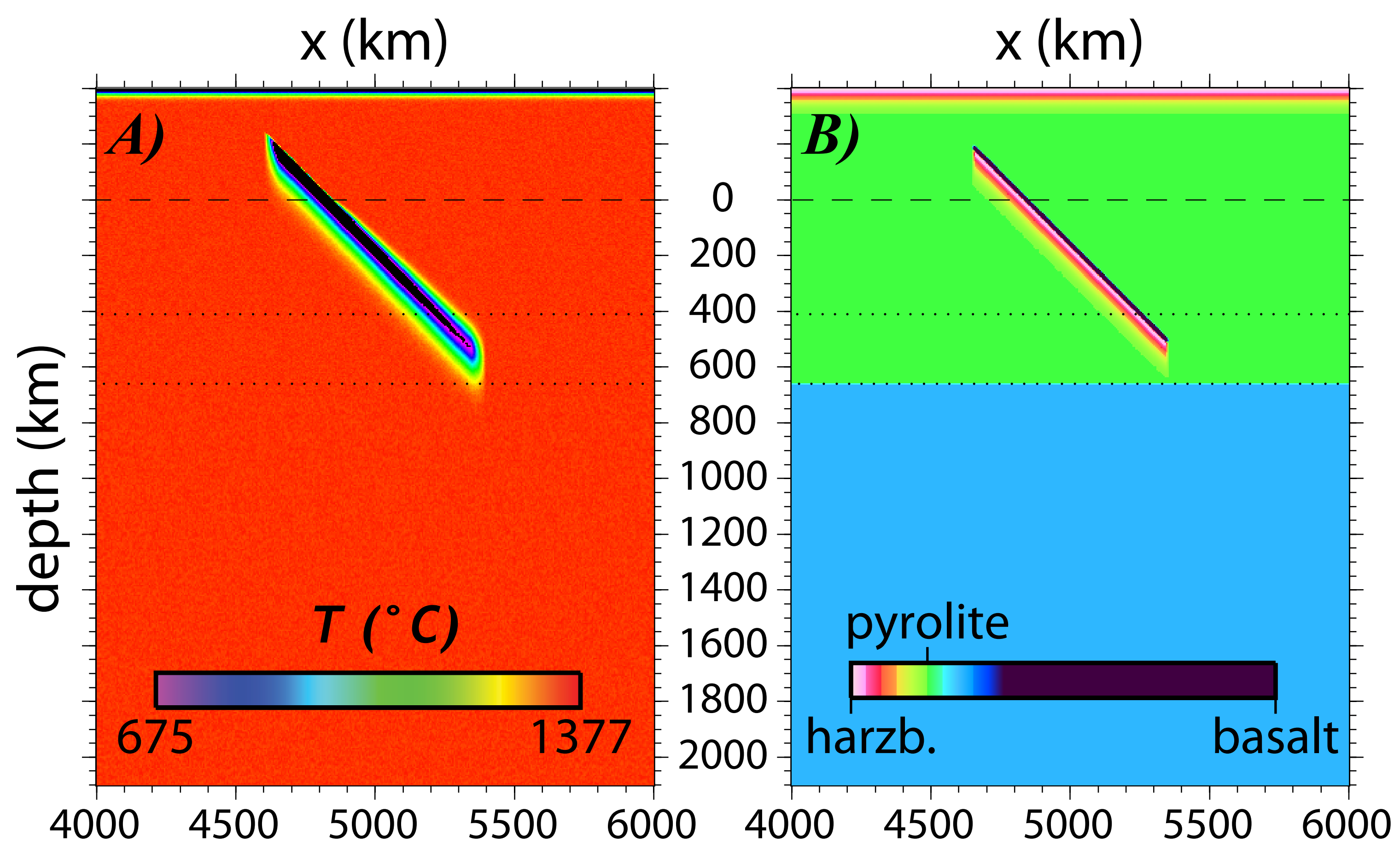
x (km)
x (km)
A)
B)
depth (km)
0
200
400
600
800
1000
1200
1400
1600
1800
2000
4000
4500
5000
5500
6000
4000
4500
5000
5500
6000
T (˚C)
675
1377
pyrolite
harzb.
basalt

Fig. S4. Initial condition of the center of the numerical-model box (note that the width of the box is 10,000 km) for a case with τ = 50 My, β = 45°, and X_{LM} = 10%. Colors denote (a) temperature and (b) composition. The part of the box above the black dashed line is the virtual extension, out of which part of the slab sinks into the model mantle. The virtual model trench is the intersection of the top of the slab with the dashed line. It retreats as the slab sinks.

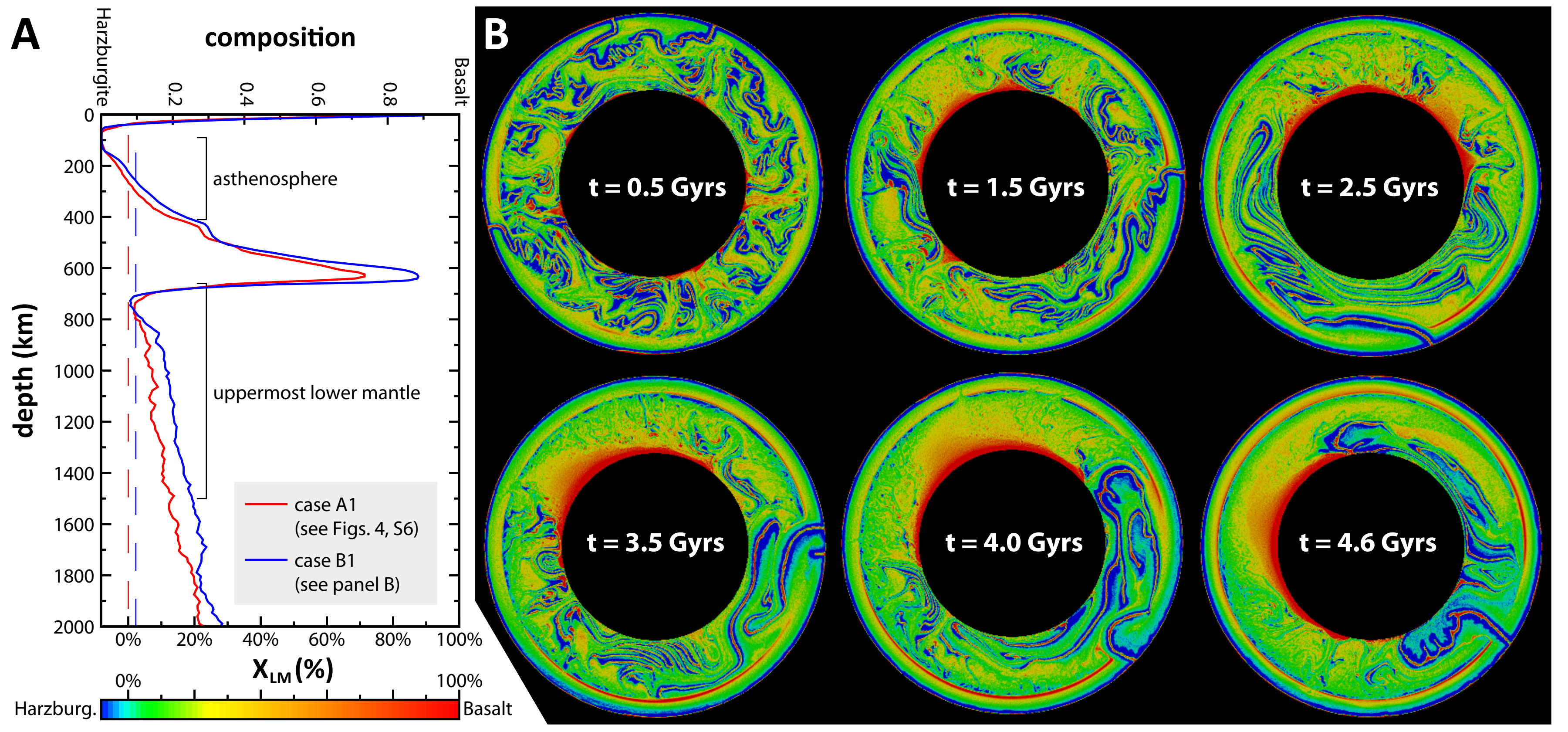

A
composition
Harzburgite
Basalt
0.2
0.4
0.6
0.8
0
200
400
600
800
1000
1200
1400
1600
1800
2000
depth (km)
asthenosphere
uppermost lower mantle
case A1
(see Figs. 4, S6)
case B1
(see panel B)
0%
20%
40%
60%
80%
100%
X_LM (%)
0%
100%
Harzburg.
Basalt
B
t = 0.5 Gyrs
t = 1.5 Gyrs
t = 2.5 Gyrs
t = 3.5 Gyrs
t = 4.0 Gyrs
t = 4.6 Gyrs

Fig. S5. Compositional mantle evolution predicted by global-scale geodynamic models for different lower-mantle density profiles of basalt. For case A1, the density anomaly of basalt is constant (1.35%) through the lower mantle. For case B1, it increases from 0.75% (at the 660) to 1.35% (at the CMB). Compositional profiles for both cases after 4.6 Gyrs are shown in (**A**). Dashed lines mark asthenospheric averages of basalt content. The compositional difference between the uppermost lower mantle and the asthenospheric average is similar for both cases. (**B**) Time-series of snapshots of composition for case B1 (see Fig. S6 for case B2).

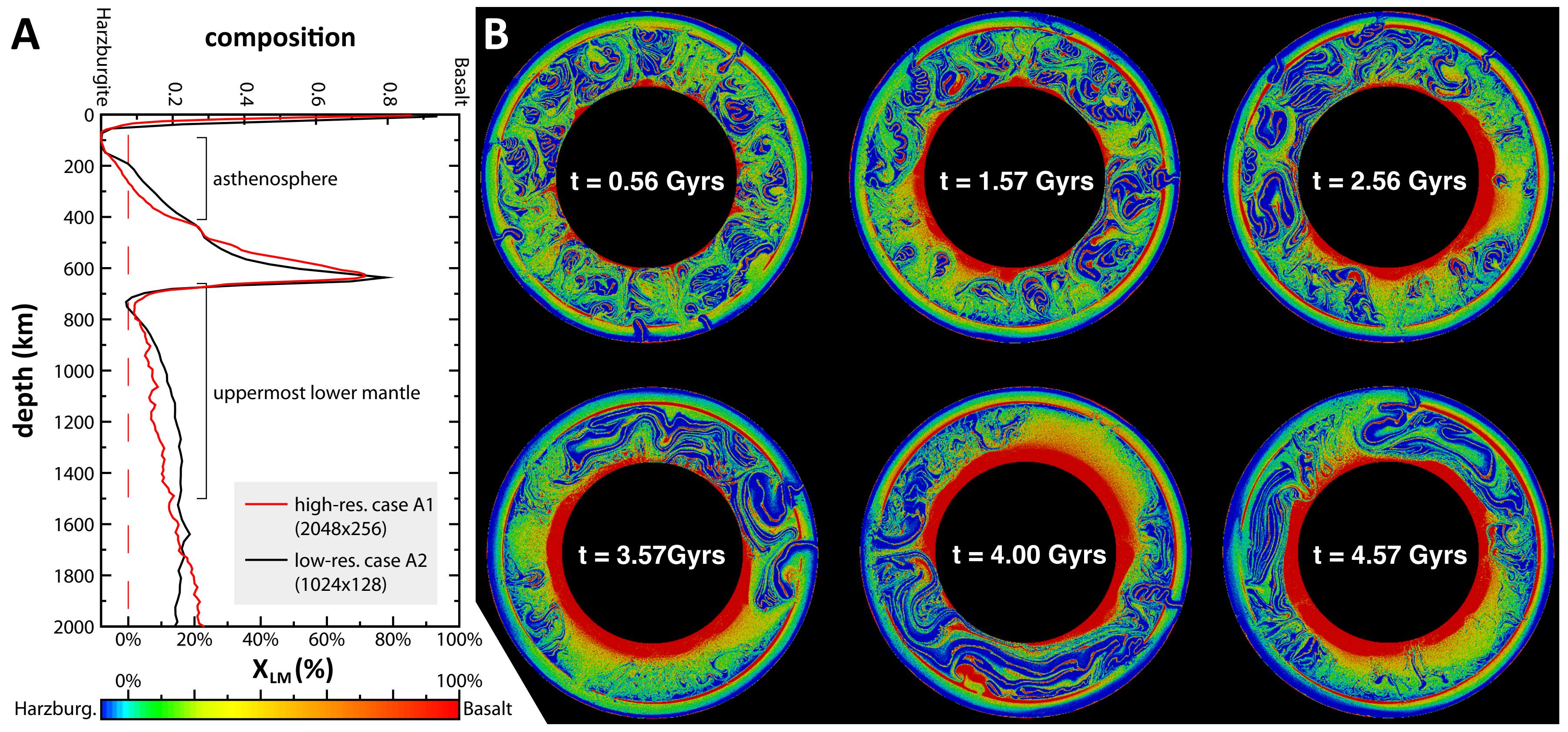

A
composition
Harzburgite
Basalt
0.2
0.4
0.6
0.8
0
200
400
600
800
1000
1200
1400
1600
1800
2000
depth (km)
asthenosphere
uppermost lower mantle
high-res. case A1
(2048x256)
low-res. case A2
(1024x128)
0%
20%
40%
60%
80%
100%
X_LM (%)
0%
100%
Harzburg.
Basalt
B
t = 0.56 Gyrs
t = 1.57 Gyrs
t = 2.56 Gyrs
t = 3.57Gyrs
t = 4.00 Gyrs
t = 4.57 Gyrs

Fig. S6. Compositional mantle evolution predicted by global-scale geodynamic models. (**A**) Compositional profile for high-resolution case A1 and low-resolution case A2 after ~4.6 Gyrs. The dashed red line marks the average asthenospheric composition for case A1 (see Figure 4a), taken as a proxy for pyrolite (X_{LM} = 0%). (**B**) Time-series of snapshots of composition for case A1.

Supplementary Tables:

Table S1: notations. The bottom four rows report the free parameters of the study (bold). ([a]) For description of depth-dependent parameters, see text (Suppl.Mat.A).

**Parameter**	**symbol**	**Value**
“real” box depth	z_{box}	2,100 km
total box depth	z^*_{box}	2,500 km
box width	x_{box}	10,000 km
mantle reference temperature	T_m	1,350 ºC
mantle reference density	ρ_m	3,300 kg/m^3
mantle reference viscosity (effective upper mantle viscosity)	η_0	4·10^{20} Pa·s
viscosity jump at 660 km depth	λ	10
activation energy	E^*	180 kJ/mol
thermal diffusivity	κ	10^{-6} m^2/s
thermal expansivity	α	[a]
density increase with depletion	$\Delta\rho_F$	[a]
basalt excess density	$\Delta\rho_X$	[a]
isochemical density jump at 660 km depth	$\Delta\rho_{660}$	300 kg/m^3
**lower mantle excess basalt content**	$\boldsymbol{X_{LM}}$	**2% to 15%**
**Clapeyron slope of the phase change at 660 km depth**	$\boldsymbol{\Gamma}$	**-0.5 to -3 MPa/K**
**initial slab dip angle**	$\boldsymbol{\beta}$	**30° to 60°**
**plate age at trench**	$\boldsymbol{T}$	**30 My to 70 My**

Table S2: Sources of data for Fig. 5.

Seismic Phase	n-Detections	% of Total	References
SdS	13	6.4	(*81-83*)
PdP	2	1.0	(*82*)
Pds	99	48.7	(*84-86*)
Sdp Scattering	6	3.0	(*87*)
Sdp Near-Source	77	37.9	(*38, 40, 41, 88-94*)
SKS-P	3	1.5	(*39*)
P'P'	3	1.5	(*95, 96*)

Table S3: Hypothetical (molar) abundances of major oxides in the lower mantle. Pyrolite and mid-ocean ridge basalt (MORB) end-member compositions are taken from ref. (*2*).

	pyrolite X_{LM} = 0%	X_{LM} = 6%	X_{LM} = 8%	X_{LM} = 10%	X_{LM} = 15%	X_{LM} = 20%	MORB X_{LM}=100%
**SiO_2**	38.71	39.49	39.75	40.01	40.67	41.32	51.75
**MgO**	49.85	47.76	47.06	46.36	44.61	42.87	14.94
**FeO**	6.17	6.22	6.24	6.26	6.30	6.35	7.06
**CaO**	2.94	3.60	3.82	4.03	4.58	5.13	13.88
**Al_2O_3**	2.22	2.70	2.86	3.02	3.42	3.81	10.19
**Na_2O**	0.11	0.23	0.28	0.32	0.42	0.52	2.18
**Mg/Si**	1.288	1.209	1.184	1.159	1.097	1.038	0.289
